# 

**Published:** 1886-07

**Authors:** 


					﻿/6 cts. a-eopy.
JULY, 1886.
$ i .00 per year.
WALES «
gJOVKNAL-J
WEALT W
ESTABLISHED 1854
°v°i- 33
Ua 7
OFFICE, 75 & 77 BARCLAY STREET, NEW YORK.
Entered as Second-class Metier at the P st-Office, New York. .
				

